# Comparative analysis of GOLPH3 expression in lymph node-positive prostate cancer: immunohistochemistry staining patterns and clinical significance

**DOI:** 10.3389/fonc.2023.1265788

**Published:** 2023-09-18

**Authors:** Paweł Kiełb, Maciej Kaczorowski, Kamil Kowalczyk, Aleksandra Piotrowska, Łukasz Nowak, Wojciech Krajewski, Adam Gurwin, Krzysztof Dudek, Piotr Dzięgiel, Agnieszka Hałoń, Tomasz Szydełko, Bartosz Małkiewicz

**Affiliations:** ^1^ University Center of Excellence in Urology, Department of Minimally Invasive and Robotic Urology, Wroclaw Medical University, Wroclaw, Poland; ^2^ Department of Clinical and Experimental Pathology, Wroclaw Medical University, Wroclaw, Poland; ^3^ Division of Histology and Embryology, Department of Human Morphology and Embryology, Wroclaw Medical University, Wroclaw, Poland; ^4^ Center for Statistical Analysis, Wroclaw Medical University, Wroclaw, Poland

**Keywords:** GOLPH3, prostate cancer, lymph nodes metastases, radical prostatectomy, golgi phosphoprotein 3

## Abstract

**Introduction:**

Prostate cancer (PCa) is the second most commonly diagnosed cancer in men worldwide. Lymph node metastasis is a poor prognostic factor for PCa. Previous studies have found that Golgi phosphoprotein 3 (GOLPH3) is overexpressed in various cancers, including PCa. We examined GOLPH3 expression in PCa cells from primary tumor and, as the first, also in metastatic lymph nodes to assess its potential as a new risk factor for PCa progression.

**Methods:**

The study included 78 patients diagnosed with lymph node-positive PCa confirmed in the postoperative material. All the patients underwent radical prostatectomy (RP) with extended lymphadenectomy. The clinical data of the patients were retrospectively analyzed, and their histopathological specimens were selected for further analysis. Immunohistochemistry (IHC) staining was performed and the expression of GOLPH3 was assessed by an experienced uropathologist using an immunoreactive scale (IRS). A correlational analysis of the obtained data with the clinicopathological data of patients was performed.

**Results:**

A positive IHC reaction for GOLPH3 was observed in all samples. IRS score for GOLPH3 expression was higher in the metastatic lymph nodes than in the prostate (not statistically significant; p=0.056). Several significant correlations were identified in connection with GOLPH3 expression levels in the prostate and metastatic lymph node tissues. No significant correlations were found between GOLPH3 expression and patient characteristics (e.g. BMI, EAU risk group, or preoperative PSA level), pathological features, or postoperative outcomes. However, we found that lymphovascular invasion (LVI) tended to be more common in patients with a higher percentage of GOLPH3-positive cells (p=0.02). We also found a positive association between the intensity of GOLPH3 staining in metastatic lymph nodes and the EAU classification. Finally, we found a significant negative correlation between the GOLPH3 expression and the efficacy of RP – the higher the expression of GOLPH3, the lower the efficacy of RP was (p<0.05).

**Conclusion:**

GOLPH3 is expressed in both prostate and metastatic lymph nodes, with higher expression in metastatic lymph nodes. High GOLPH3 expression was associated with the occurrence of LVI, higher-risk group in the EAU classification, and lower efficacy of the RP, but there was no significant correlation with other pathological features or postoperative outcomes.

## 1 Introduction

Prostate cancer (PCa) is the second most commonly diagnosed cancer in men worldwide, and is one of the leading causes of cancer-related deaths in the male population (1). The incidence of PCa increases with the age of patients; therefore, due to increasing life expectancy, there will be more patients with PCa. This will be an even more significant health problem in society than it is today (2). Due to the dynamic development of methods for the diagnosis and treatment of PCa, the results of treatment of patients are gradually improving. Despite the presence of constantly improving therapy protocols, choosing the best treatment plan for a given patient is difficult, and the final effect is uncertain. This is due to the fact that there is still a lack of more precise tools to accurately assess the survival prognosis and the risk of progression or metastasis after primary treatment of PCa.

Differences in the treatment effects between patients with PCa may be related to the high heterogeneity of prostate tumors, which may affect the effectiveness of primary or adjuvant therapy. Numerous studies suggest that the analysis of the expression of immunohistochemical (IHC) markers in the tissues of patients with PCa, such as Golgi phosphoprotein 3 (GOLPH3), may be an important tool for improving diagnosis, assessing prognosis, risk of progression, and potential effects of primary treatment or response to adjuvant treatment (3–7). In addition, they can be a valuable supplement to the already used classic prognostic factors, such as prostate specific antigen (PSA) level, clinical stage, or histological grade, determined on the basis of prostate biopsy results. Despite the promising results of these studies, analysis of the expression of these markers is not routinely recommended by the guidelines.

The presence of lymph node metastases is an important risk factor with a decidedly negative impact on survival and the risk of recurrence after primary treatment in patients with PCa. Nodal metastases also affect the therapeutic process in patients through the selection of adequate adjuvant treatment and more rigorous follow-up after primary treatment (8, 9). Despite continuous intensive technological developments, the assessment of nodal involvement using radiological imaging techniques remains inferior to lymphadenectomy (10, 11). Currently, the gold standard for detecting dissemination to the lymph nodes is extended pelvic lymphadenectomy during radical prostatectomy (RP). However, this is an additional invasive procedure that does not bring survival benefits and significantly increases the risk of treatment side effects, such as increased blood loss, longer surgery and hospitalization time, and an increased risk of lymphocele development in the postoperative period (12). In the absence of more accurate methods to determine lymph node status, extended pelvic lymphadenectomy should be performed in intermediate- and high-risk PCa patients (13).

In this study, we investigated the GOLPH3 protein, which performs key functions in the Golgi apparatus, such as maintaining the ribbon structure and its glycosylation, as well as intracellular vesicular transport (14–16). GOLPH3 was the first Golgi oncoprotein to be described (17). Its pro-tumor effects are complex, and a thorough understanding of all these mechanisms requires further research. To date, several possible pathways using GOLPH3 in the process of carcinogenesis have been proposed, including increased transport from the Golgi apparatus to the plasma membrane, disruption of genome structure stability, disorganization of endocytosis regulation, and changes in glycosylation of proteins in the Golgi apparatus (16). The oncogenic effect of GOLPH3 and its impact on the course of the disease have been demonstrated in studies on melanoma, colon adenocarcinoma, glioblastoma, and non-small-cell lung cancer (18).

The precise mechanism of GOLPH3 oncogenic effect in PCa pathogenesis is unknown. Several modes of action have been proposed based on currently available studies. One of these mechanisms is the activation of the mammalian target of rapamycin (mTOR) signalling pathway, which stimulates the activity of the kinase B protein while decreasing the transcriptional activity of the forkhead box protein O gene (3, 18–20). Furthermore, the effect of mTOR activation on cell differentiation suggests a significant role in the transition from hormone-sensitive to hormone-refractory PCa (6). Another suggested mechanism is GOLPH3 stimulating effect on matrix metalloproteinases 9 (MMP9) secretion in PCa cells via epidermal growth factor receptor (EGFR) and Src kinase, which appear to be important, especially for the formation of PCa metastases (4, 21–26).

Despite the results suggesting a correlation between GOLPH3 and malignant tumor progression, to date, no studies have assessed the expression of GOLPH3 in lymph nodes. In our study, we comprehensively investigated GOLPH3 expression in PCa cells from primary tumor tissues and metastatic lymph nodes. The evaluation of GOLPH3 expression in metastatic lymph nodes has not been previously reported, making our study unique in this regard. We correlated the obtained results with clinical data of patients with lymph node metastases to assess the application of GOLPH3 as a new negative risk factor for PCa progression.

## 2 Materials and methods

### 2.1 Patients and pathological specimens

The study included 78 patients with diagnosed PCa, in whom metastases in the lymph nodes were detected in the postoperative material. All patients underwent RP with extended lymphadenectomy between January 2012 and September 2018 at the University Urology Center (Wrocław, Poland). We retrospectively analyzed the clinical data of the patients included in the study and selected their histopathological specimens obtained after prostatectomy for further analysis. The obtained material was evaluated by an experienced uropathologist. Tumor stage and grade ware assessed according to the 2017 Tumour, Node, Metastasis (TNM) classification of PCa and the Gleason system. In addition, classifications such as International Society of Urological Pathology (ISUP) 2014 grade (group) system and European Association of Urology (EAU) risk groups for biochemical recurrence of localized and locally-advanced PCa were used to better characterize patients. Efficacy of RP was defined as a PSA level <0.1 ng/ml at the first measurement after RP, usually 6 weeks after surgery.

### 2.2 Tissue microarrays

**Figure 1:**
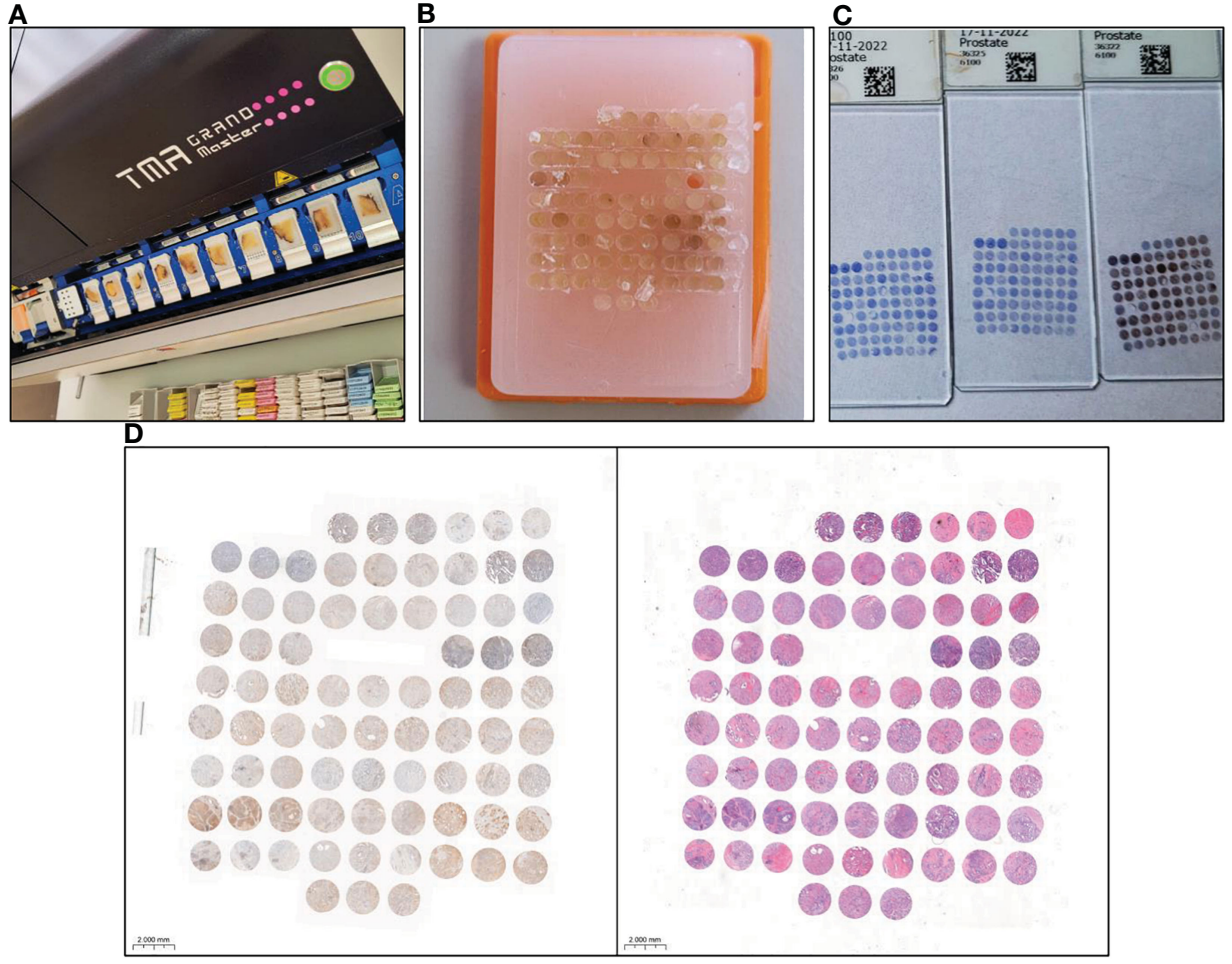
Tissue microarray (TMA) preparation and immunohistochemical (IHC) staining for GOLPH3 expression analysis. **(A)** Tissue microarrayer TMA Grand Master (3DHISTECH Ltd.), with selected donor blocks containing prostate tissue. **(B)** Finished tissue microarray. **(C)** Slides after IHC staining of prostate tissue prepared for scanning. **(D)** The result of scanning a single slide in the program is ready for evaluation of GOLPH3 expression in prostate tissue. The tissue on the left side was IHC stained. On the right side, the same material was stained with haematoxylin and eosin **(HE)** to aid the evaluation.

The tissue microarrays (TMAs) technique is widely recognized as a valid approach for preserving material in paraffin blocks, offering numerous benefits such as cost-effectiveness, consistent IHC reaction conditions, and efficient evaluation of IHC results, with only minor limitations (27, 28). In our study 8 TMAs were prepared. Prior to performing TMAs blocks the histological slides stained with hematoxylin and eosin (HE) were obtained from whole samples of prostate and lymph nodes with detected prostate adenocarcinoma cells archived in the form of paraffin blocks (donor blocks). The slides were scanned using the Pannoramic Midi II histological scanner (3DHISTECH Ltd.). After that by using the Panoramic Viewer Program (3DHISTECH Ltd.), the representative areas from the entire sections where selected by uropathologist. In addition, to increase the representativeness of each case, 3 representative cores with a size of 1.5 mm from the donor block were selected and then transferred to the TMA ‘recipient’ block using the TMA Grand Master (3DHISTECH Ltd.). The TMA creation process is presented in **Figure 1**.

### 2.3 Immunohistochemistry

IHC reactions were performed on 4 μm paraffin sections obtained from TMA blocks using an automated staining platform, Autostainer Link48 (Dako, Glostrup, Denmark). First, the slides were deparaffinized, rehydrated, and antigen retrieval was performed by boiling the sections in EnVision FLEX Target Retrieval Solution, High pH (97°C, 20 min; pH 9) in PTLink (Dako). Endogenous peroxidase activity was blocked by incubation for 5 min with the EnVision FLEX Peroxidase-Blocking Reagent (Dako). Monoclonal mouse anti-GOLPH3 antibody (1:2000; cat. No. LS-B5044, LS Bio, Lynnwood, DC, USA) was used as the primary antibody (20 min incubation), followed by incubation with a secondary antibody conjugated with horseradish peroxidase (EnVision FLEX/HRP, 20 min incubation). Subsequently, 3,3-diaminobenzidine was applied and the sections were incubated for 10 min at RT. All sections were counterstained with EnVision FLEX Hematoxylin (Dako) for 5 min. After dehydration in graded ethanol concentrations (70%, 96%, absolute) and xylene, all slides were closed with coverslips in SUB-X Mounting Medium. The slides were scanned using a histologic scanner, Pannoramic MIDI (3DHistech). Reactions were evaluated with the use of Quant Center software (3DHistech) under researcher supervision. In order to evaluate the expression of GOLPH3, for every case, six TMA cores (3 from prostate and 3 from metastatic lymph node) were assessed using a Pannoramic Viewer Digital image analysis. The expression assessment of GOLPH3 was performed by an experienced uropathologist unaware of detailed patient clinical information, by using immunoreactive scale (IRS) by Remmele and Stegner (29, 30), presented in **Table 1**.

**Table 1:** Immunoreactive scale (IRS) by Remmele and Stegner.

Immunoreactive Scale (IRS)					
A – Percentage of positive cancer cells		B – Staining intensity			
Score		Score			
0	no cells with positive reaction	0		no colour reaction
1	< 10% cells with positive reaction	1		mild reaction
2	10–50% cells with positive reaction	2		moderate reaction
3	51–80% cells with positive reaction	3		intense reaction
4	> 80% cells with positive reaction			
IRS SCORE (A X B): 0-12 points					
Final score		Level of expression			
1-7		Low expression			
8-12		High expression			

IRS score taking into account the percentage of positively stained prostate cancer cells (A) and the intensity of staining (B) and final score is the result of multiplying these values (A X B). Based on the IRS score, patients were divided into a group of low and high GOLPH3 expression as presented.

**Figure 2:**
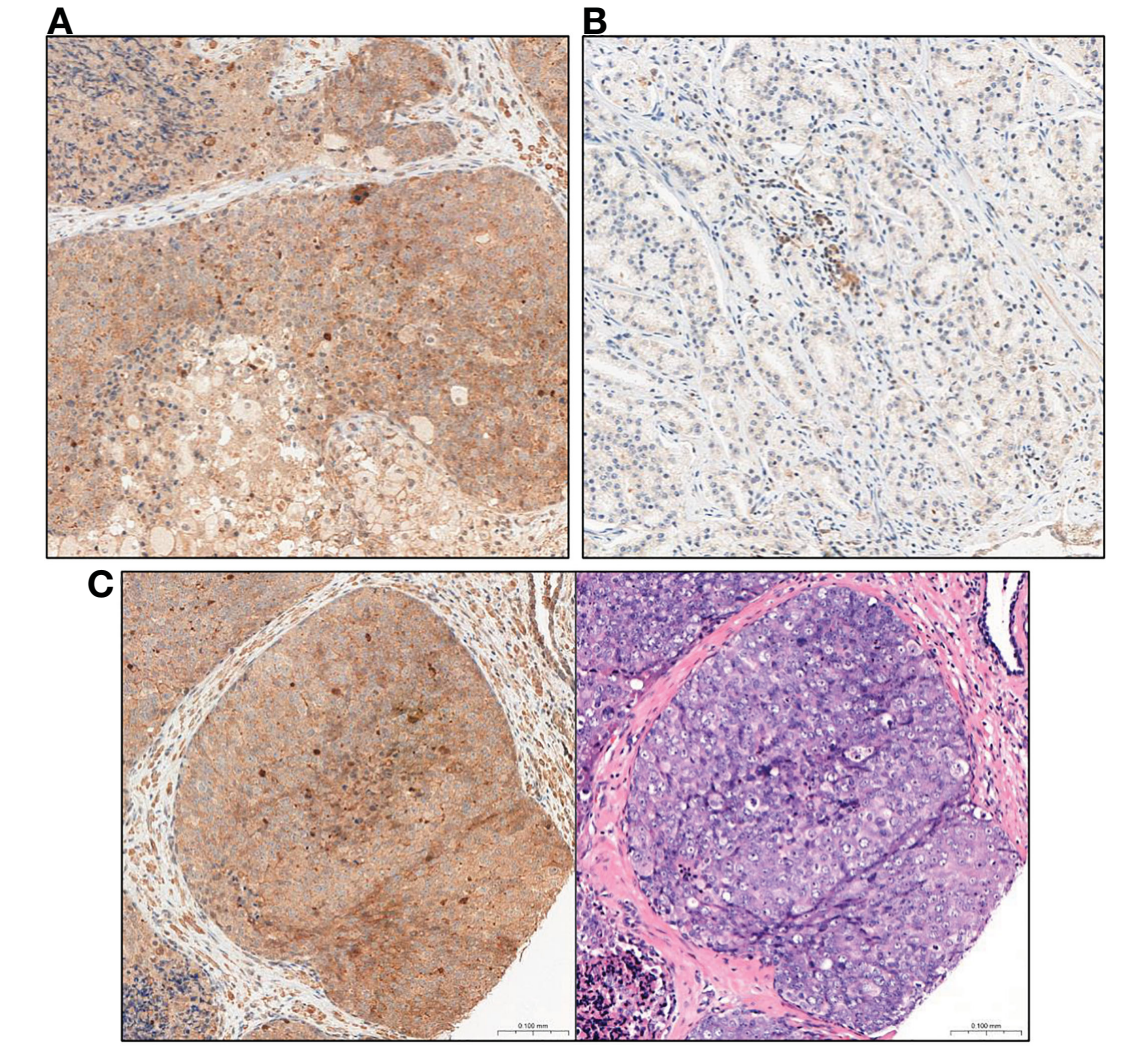
Comparison of GOLPH3 expression in prostate tissue **(A)** High GOLPH3 expression in prostate tissue. **(B)** Low GOLPH3 expression in the prostate tissue. **(C)** Comparison of the image after immunohistochemical staining with high expression of GOLPH3 in the prostate tissue and the same tissue stained only with haematoxylin and eosin (HE). Magnification, x15.

**Figure 3:**
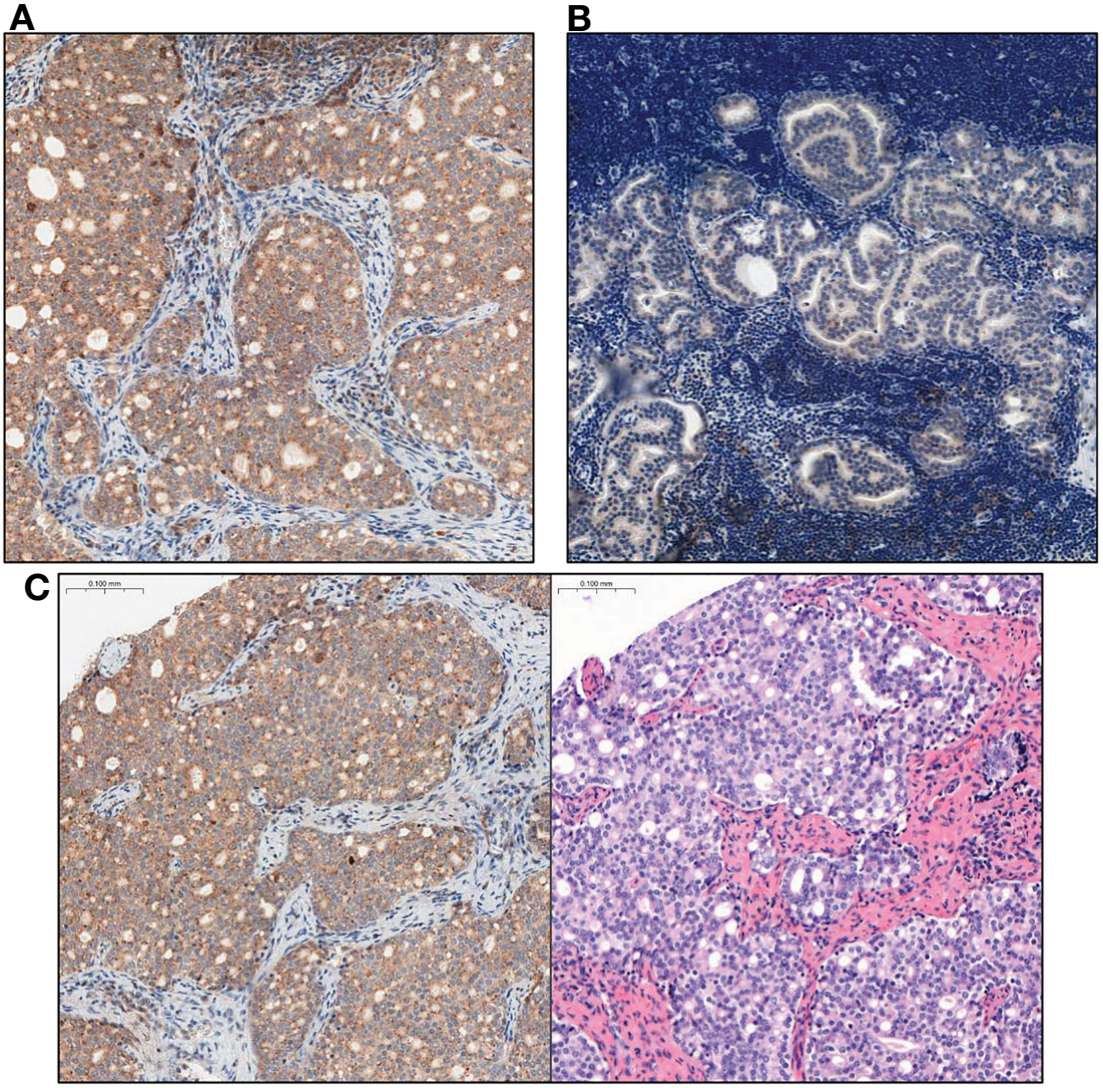
Comparison of GOLPH3 expression in lymph nodes with prostate cancer metastasis. **(A)** High GOLPH3 expression in the metastatic lymph node tissues. **(B)** Low GOLPH3 expression in the metastatic lymph node tissues. **(C)** Comparison of the image after immunohistochemical staining with high expression of GOLPH3 in metastatic lymph node tissue and the same tissue stained only with haematoxylin and eosin (HE). Magnification, x15.

In short, IRS score taking into account the percentage of positively stained PCa cells (A) and the intensity of staining (B) and final score is the result of multiplying these values (A X B). Material from the prostate and the metastatic lymph node were assessed separately for each patient. The final IRS score for prostate and metastatic lymph node was the average score obtained from the assessment of each of the 3 cores of a given tissue type. The figures show a comparison of the intensity of GOLPH3 expression in the evaluated prostate preparations (**Figure 2**) and metastatic lymph nodes (**Figure 3**).

### 2.4 Statistical analysis

The mean, standard deviation (SD), minimum (Min), maximum (Max), median (Me), lower quartile (Q1), and upper quartile (Q3) for quantitative variables were calculated. The Kolmogorov-Smirnov and Shapiro-Wilk tests were used to confirm that quantitative variables’ empirical distribution fits to a normal distribution. To evaluate the connection between monotonic relationships between variables, Spearman’s rank correlation coefficient was determined. In contingency tables, qualitative (nominal and categorical) variables were presented as numbers (n) and percentages (%). The Mann-Whitney U test was applied to verify the significance of differences in quantitative parameters between the two groups, and Pearson’s Chi squared test was used to confirm the independence of two qualitative factors. In all analyzed cases, the associations were considered statistically significant for p < 0.05. All statistical analyses were performed using Statistica v.13.3 (TIBCO Software Inc., Palo Alto, CA, USA).

## 3 Results

General characteristics of the patients presented in **Table 2**.

**Table 2:** General characteristics and clinicopathological parameters of the patients.

Variable	Statistics
General characteristics of patients	
Age (years):	
*M ± SD*	65.0 ± 5.6
BMI (kg/m^2^):	
*M* ± *SD*	28.1 ± 3.6
Preoperative PSA (ng/ml):	
*Me* [*Q*1; *Q*3]	19.7 (10.8; 36.1)
EAU risk group, n (%):	
Low-risk Intermediate-risk High-risk High-risk locally advanced	1 (1.3) 8 (10.3) 39 (50.0) 30 (38.5)
Clinicopathological parameters	
pT, n (%):	
2a 2c 3a 3b	1 (1.3) 9 (11.5) 14 (17.9) 54 (69.2)
Postoperative Gleason, n (%):	
3 + 3 3 + 4 3 + 5 4 + 3 4 + 4 4 + 5 5 + 3 5 + 4 5 + 5	1 (1.3) 11 (14.1) 4 (5.1) 19 (24.4) 3 (3.8) 29 (37.2) 2 (2.6) 8 (10.3) 1 (1.3)
Postoperative GGG ISUP, n (%):	
1 2 3 4 5	1 (1.3) 11 (14.1) 19 (24.4) 9 (11.5) 38 (48.7)
Extracapsular extension of prostate, n (%):	
Yes No	67 (85.9) 11 (14.1)
Resection margin, n (%):	
Positive Negative	55 (70.5) 23 (29.5)
Neurovascular invasion, n (%):	
Yes No No data	71 (91.0) 1 (1.3) 6 (7.7)
Lymphovascular invasion, n (%):	
Yes No No data	57 (73.1) 16 (20.5) 5 (6.4)
Affected lymph nodes (%):	
*Me* [*Q*1; *Q*3]	12.1 (8.0; 27.3)
Efficacy of RP, n (%):	
Yes No	37 (47.4) 41 (52,6)

M, arithmetic mean; SD, standard deviation; BMI, body mass index; PSA, prostate specific antigen; Me, median; Q1, lower quartile; Q3, upper quartile; EAU, European Association of Urology; n, number; %, percentage; pT, pathological tumor stage; GGG ISUP, International Society of Urological Pathology (ISUP) 2014 grade (group) system; efficacy of RP, defined as an PSA level <0.1 ng/ml at the first measurement after radical prostatectomy.

In all the analyzed tissue samples, we found a positive immunohistochemical reaction in PCa cells confirming the expression of GOLPH3 in the analyzed material.

The level of GOLPH3 expression, assessed using the IRS scale, was higher in the material from the metastatic lymph node than from the prostate (IRS score: 8 vs. 6 score; p=0.056). However, the statistically significant difference between prostate and metastatic lymph nodes was only in the percentage of GOLPH3 positive cancer cells found in the evaluated tissue sample (A: 4 vs. 3; p=0.046), with no significant difference in the intensity of staining (p=0.278).

Using a simplified GOLPH3 expression level classification based on the IRS score, most prostate samples were found to have low GOLPH3 expression levels, whereas metastatic lymph node material was found to have high GOLPH3 expression. However, these differences were not statistically significant (p=0.148). The results of the statistical analysis are presented in **Table 3**.

**Table 3:** Basic descriptive statistics of the evaluation of GOLPH3 expression in prostate and metastatic lymph node tissues and the results of comparisons (N = 78).

GOLPH3 expression (IRS scale)	Prostate	Metastatic lymph node	p-value
**A - Percentage of GOLPH3 positive cancer cells (score)**			**0.046**
***Me* [*Q*1; *Q*3]**	3 [3; 4]	4 [3; 4]	
***Min* - *Max***	2 - 4	2 - 4	
**B - Intensity of staining (score)**			0.278
***Me* [*Q*1; *Q*3]**	2 [1; 2]	2 [2; 2]	
***Min* - *Max***	1 - 3	1 - 3	
**IRS score (A × B)**			0.056
***Me* [*Q*1; *Q*3]**	6 [4; 8]	8 [6; 8]	
***Min* - *Max***	2 - 12	2 – 12	
**GOLPH3 expression level:**			0.148
**Low expression (1 – 7 score), n (%)**	47 (60.3)	37 (47.4)	
**High expression (8 – 12 score), n (%)**	31 (39.7)	41 (52.6)	

IRS, immunoreactive scale; A, percentage of positive cancer cells (value from IRS scale); B, staining intensity (value from IRS scale); Me, median; Q1, lower quartile; Q3, upper quartile; Min, minimum; Max, maximum; n, number; %, percentage.

**Figure 4:**
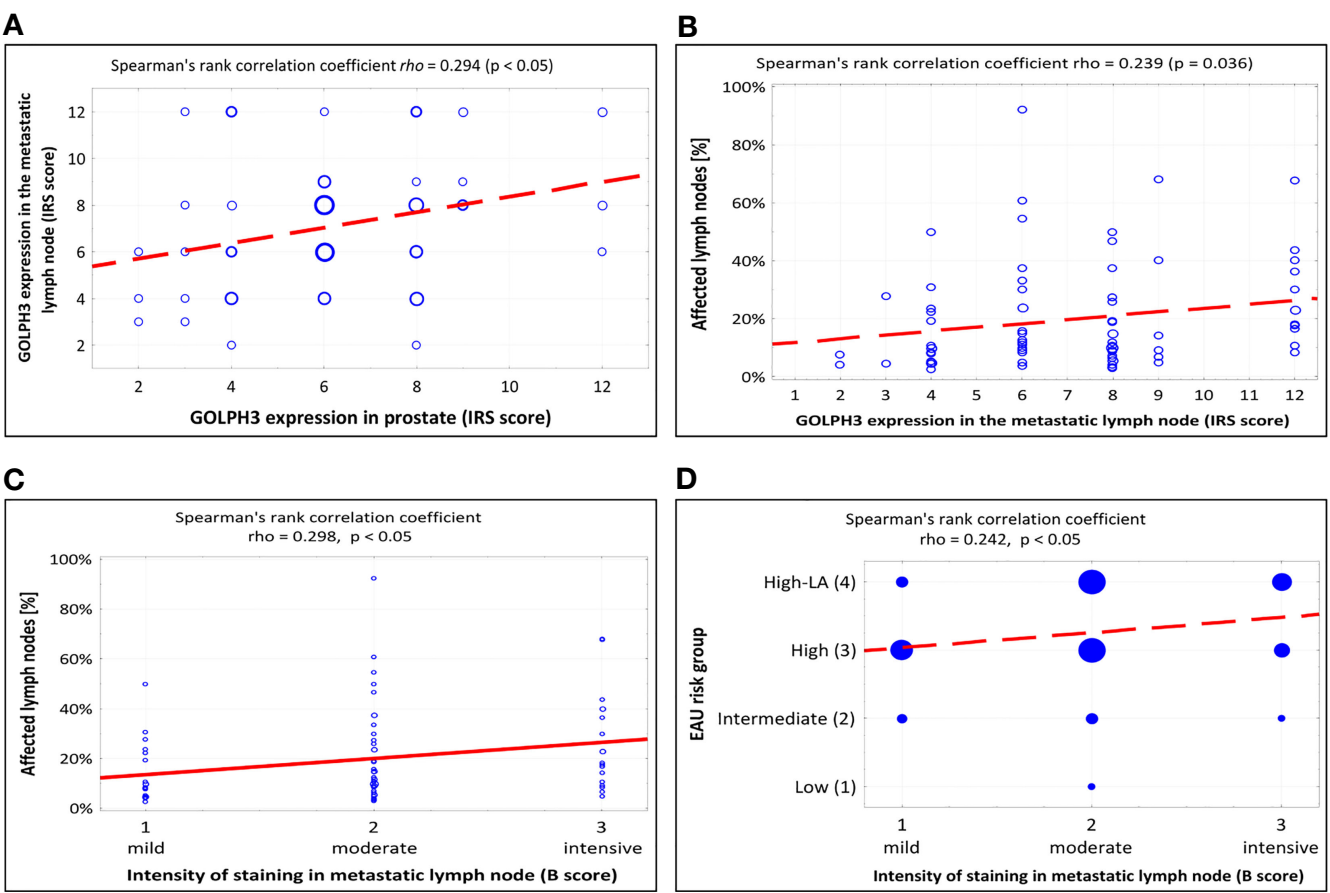
Summary of scatterplots and Spearman rank correlation coefficients. **(A)** Correlation between GOLPH3 expression in the metastatic lymph node and GOLPH3 expression in prostate (IRS score). **(B)** Correlation between the percentage of affected lymph nodes and GOLPH3 expression in the metastatic lymph node (IRS score). **(C)** Correlation between the percentage of affected lymph nodes and intensity of staining in metastatic lymph node (B-score in IRS scale). **(D)** Correlation between the EAU risk group and intensity of staining in metastatic lymph node (B-score in IRS scale). IRS - immunoreactive scale, EAU - European Association of Urology.

A significant positive correlation was found between the level of GOLHP3 expression in prostate and in the metastatic lymph node (rho=0,294, p<0.05; **Figure 4A**).

There were no significant correlations between the level of GOLPH3 expression (expressed by IRS score) in the prostate or metastatic lymph nodes and the patients’ age, BMI, EAU risk group, postoperative GGG ISUP, or preoperative PSA level.

A significant positive correlation was found between the level of GOLPH3 expression in metastatic lymph nodes and the percentage of affected lymph nodes (p=0.036, **Figure 4B**). **Table 4** shows the results of the statistical analysis.

**Table 4:** Correlation analysis between GOLPH3 expression in prostate and metastatic lymph node assessed in IRS score and quantitative variables.

	Prostate		Metastatic lymph node	
	rho	p	rho	p
**Preoperative PSA (ng/ml)**	-0.016	0.885	0.100	0.380
**Affected lymph nodes (%)**	0.137	0.230	0.239	0.036
**Age (years)**	-0.121	0.286	-0.030	0.795
**BMI (kg/m^2^)**	0.126	0.271	-0.139	0.223
**EAU risk group**	0.045	0.692	0.183	0.109
**Postoperative GGG ISUP**	0.002	0.989	0.055	0.632

BMI, body mass index; PSA, prostate specific antigen; EAU, European Association of Urology; %, percentage; GGG ISUP, International Society of Urological Pathology (ISUP) 2014 grade (group) system.

No statistically significant correlation was found between the level of GOLPH3 expression in prostate and metastatic lymph nodes (assessed based on the IRS score) and the pathological features or postoperative outcomes of patients. **Table 5** contains the results of the statistical analysis.

**Table 5:** Number (percentage) of patients in groups differing in the level of GOLPH3 expression (based on IRS score) in the material from the prostate or metastatic lymph node, risk factors, and results of tests of independence.

GOLPH3 expression level (IRS score based)							
Variables		Expression of GOLPH3 in PROSTATE			Expression of GOLPH3 in METASTATIC LYMPH NODE		
		Level of expression		p-value	Level of expression		p-value
		High N = 31	Low N = 47		High N = 41	Low N = 37	
		n (%)	n (%)		n (%)	n (%)	
**pT**	3a and 3b	30 (96.7)	38 (80.8)	0.087	35 (85.4)	33 (89.2)	0.869
	2a and 2c	1 (3.2)	9 (15.2)		6 (14.6)	4 (10.8)	
**ECE of prostate**	Yes	28 (90.3)	39 (83.0)	0.511	33 (80.5)	34 (91.9)	0.199
	No	3 (9.7)	8 (17.0)		8 (19.5)	3 (8.1)	
**Resection margin**	Positive	22 (71.0)	33 (70.2)	0.855	28 (68.3)	27 (73.0)	0.804
	Negative	9 (29.0)	14 (29.8)		13 (31.7)	10 (27.0)	
**ECE of lymph node**	Yes	6 (19.4)	13 (27.7)	0.435	11 (26.8)	8 (21.6)	0.610
	No	25 (80.6)	34 (72.3)		30 (73.2)	29 (78.4)	
**NVI**	Yes	27 (100.0)	44 (97.8)	1.000	38 (100.0)	33 (97.1)	0.472
	No	0 (0.0)	1 (2.2)		0 (0.0)	1 (2.9)	
**LVI**	Yes	22 (78.6)	35 (77.8)	1.000	31 (83.8)	26 (72.2)	0.269
	No	6 (21.4)	10 (22.2)		6 (16.2)	10 (27.8)	
**Efficacy of RP**	Yes	12 (48.0)	25 (61.0)	0.321	20 (62.5)	17 (50.0)	0.332
	No	13 (52.0)	16 (39.0)		12 (37.5)	17 (50.0)	
**Expression of GOLPH3 in metastatic lymph node**	High	20 (64.5)	21 (44.7)	0.107	XX	XX	XX
	Low	11 (35.5)	26 (55.3)		XX	XX	
**Expression of GOLPH3 in prostate**	High	XX	XX	XX	20 (48.8)	11 (29.7)	0.107
	Low	XX	XX		21 (51.2)	26 (70.3)	

IRS, immunoreactive scale; n, number; %, percentage; pT, pathological tumor stage; ECE, extracapsular extension; NVI, neurovascular invasion; LVI, lymphovascular invasion; efficacy of RP, defined as an PSA level <0.1 ng/ml at the first measurement after radical prostatectomy.

However, when analyzing the relationship between these features and the percentage of GOLPH3 positive cancer cells (“A” score in IRS scale) in the prostate and metastatic lymph node samples, a significant correlation was found with the efficacy of the RP - the efficacy of RP was higher in patients in whom the percentage of GOLPH3 positive cancer cells in prostate was lower (75% vs. 36.7% for 51-80% GOLPH3 positive cells and >80% GOLPH3 positive cells respectively; p=0.001). In addition, a statistically significant correlation was found between the percentage of GOLPH3 positive cancer cells in metastatic lymph nodes and the occurrence of lymphovascular invasion (LVI) (p=0.02). LVI was significantly less common in patients with a percentage of GOLPH3 positive cancer cells between 11-50% than 51-80% (0% vs. 75%; p=0.026) and >80% (0% vs. 83%; p=0.004). The results of this analysis are summarized in **Table 6**.

**Table 6:** Number (percentage) of patients in groups differing in the percentage of GOLPH3 positive prostate cancer cells (“A” score in IRS scale) in the material from the prostate or metastatic lymph node, risk factors, and results of chi-square tests of independence.

Percentage of GOLPH3 positive prostate cancer cells (“A” score in IRS scale)															
Variables		Percentage of GOLPH3 positive prostate cancer cells in PROSTATE							Percentage of GOLPH3 positive prostate cancer cells in METASTATIC LYMPH NODE						
		A score (% ranges of GOLPH3 positive cancer cells)						p-value	A score (% ranges of GOLPH3 positive cancer cells)						p-value
		2 (11-50%) N = 4		3 (51-80%) N = 37		4 (>80%) N = 37			2 (11-50%) N = 2		3 (51-80%) N = 26		4 (>80%) N = 50		
		n	(%)	n	(%)	n	(%)		n	(%)	n	(%)	n	(%)	
**pT**	3a and 3b	3	75.0	30	81.1	35	94.6	0.167	2	100.0	22	84.6	44	88.0	0.788
	2a and 2c	1	25.0	7	18.9	2	5.4		0	0.0	4	15.4	6	12.0	
**ECE of prostate**	Yes	3	75.0	30	81.1	34	91.9	0.333	2	100.0	23	88.5	42	84.0	0.734
	No	1	25.0	7	18.9	3	8.1		0	0.0	3	11.5	8	16.0	
**Resection margin**	Positive	2	50.0	24	64.9	29	78.4	0.290	2	100.0	16	61.5	37	74.0	0.344
	Negative	2	50.0	13	35.1	8	21.6		0	0.0	10	38.5	13	26.0	
**ECE of lymph node**	Yes	1	25.0	10	27.0	8	21.6	0.863	0	0.0	8	30.8	11	22.0	0.503
	No	3	75.0	27	73.0	29	78.4		2	100.0	18	69.2	39	78.0	
**NVI**	Yes	4	100.0	34	97.1	33	100.0	0.585	1	100.0	23	95.8	47	100.0	0.363
	No	0	0.0	1	2.9	0	0.0		0	0.0	1	4.2	0	0.0	
**LVI**	Yes	4	100.0	26	74.3	27	79.4	0.484	0	0.0	18	75.0	39	83.0	**0.020**
	No	0	0.0	9	25.7	7	20.6		2	100.0	6	25.0	8	17.0	
**Efficacy of RP**	Yes	2	50.0	24	75.0	11	36.7	**0.010**	2	100.0	11	44.0	24	61.5	0.172
	No	2	50.0	8	25.0	19	63.3		0	0.0	14	56.0	15	38.5	
**Expression of GOLPH3 in metastatic lymph node**	High	0	0.0	22	59.5	19	51.4	**0.035**	XX	XX	XX	XX	XX	XX	XX
	Low	4	100.0	15	40.5	18	48.6		XX	XX	XX	XX	XX	XX	
**Expression of GOLPH3 in prostate**	High	XX	XX	XX	XX	XX	XX	XX	1	50.0	7	26.9	23	46.0	0.261
	Low	XX	XX	XX	XX	XX	XX		1	50.0	19	73.1	27	54.0	

IRS, immunoreactive scale; n, number; %, percentage; pT, pathological tumor stage; ECE, extracapsular extension; NVI, neurovascular invasion; LVI, lymphovascular invasion; efficacy of RP, defined as an PSA level <0.1 ng/ml at the first measurement after radical prostatectomy.

We also found a statistically significant relationship related to the efficacy of RP, similar to that already described, between the intensity of staining GOLPH3 positive cancer cells (“B” score in IRS scale) in metastatic lymph node (p=0.026). The efficacy of RP was higher in patients with moderate staining intensity than in those with intensive staining (68.4% vs. 25%; p=0.002). The statistical analysis is shown in **Table 7**. Additionally, we also found a positive correlation between the intensity of GOLPH3 staining (“B” score in IRS scale) in the metastatic lymph nodes and the percentage of all metastatic lymph nodes (rho=0.298, p<0.05; **Figure 4C**) and also the EAU classification (rho=0.242, p<0.05; **Figure 4D**).

**Table 7:** Number (percentage) of patients in groups differing in the intensity of staining in GOLPH3 positive prostate cancer cells (“B” score in IRS scale) in the material from the prostate or metastatic lymph node, risk factors, and results of chi-square tests of independence.

Intensity of staining in GOLPH3 positive prostate cancer cells (“B” score in IRS scale)															
Variables		Intensity of staining in GOLPH3 positive prostate cancer cells in PROSTATE							Intensity of staining in GOLPH3 positive prostate cancer cells in METASTATIC LYMPH NODE						
		B score (staining intensity)						**p-value**	B score (staining intensity)						**p-value**
		**1** (mild) N = 20		**2** (moderate) N = 47		**3** (intensive) N = 11			**1** (mild) N = 19		**2** (moderate) N = 41		**3** (intensive) N = 18		
		n	(%)	n	(%)	n	(%)		n	(%)	n	(%)	n	(%)	
**pT**	3a and 3b	18	90.0	39	83.0	11	100.0	0.286	18	94.7	33	80.5	17	94.4	0.177
	2a and 2c	2	10.0	8	17.0	0	0.0		1	5.3	8	19.5	1	5.6	
**ECE of prostate**	Yes	18	90.0	39	83.0	10	90.9	0.658	17	89.5	34	82.9	16	88.9	0.729
	No	2	10.0	8	17.0	1	9.1		2	10.5	7	17.1	2	11.1	
**Resection margin**	Positive	15	75.0	32	68.1	8	72.7	0.838	16	84.2	27	65.9	12	66.7	0.321
	Negative	5	25.0	15	31.9	3	27.3		3	15.8	14	34.1	6	33.3	
**ECE of lymph node**	Yes	6	30.0	12	25.5	1	9.1	0.412	3	15.8	9	22.0	7	38.9	0.229
	No	14	70.0	35	74.5	10	90.9		16	84.2	32	78.0	11	61.1	
**NVI**	Yes	18	94.7	43	100.0	10	100.0	0.243	16	94.1	39	100.0	16	100.0	0.194
	No	1	5.3	0	0.0	0	0.0		1	5.9	0	0.0	0	0.0	
**LVI**	Yes	16	84.2	34	77.3	7	70.0	0.665	12	66.7	32	80.0	13	86.7	0.350
	No	3	15.8	10	22.7	3	30.0		6	33.3	8	20.0	2	13.3	
**Efficacy of RP**	Yes	7	41.2	25	61.0	5	62.5	0.356	8	50.0	26	68.4	3	25.0	**0.026**
	No	10	58.8	16	39.0	3	37.5		8	50.0	12	31.6	9	75.0	
**Expression of GOLPH3 in metastatic lymph node**	High	7	35.0	24	51.1	10	90.9	**0.011**	XX	XX	XX	XX	XX	XX	XX
	Low	13	65.0	23	48.9	1	9.1%		XX	XX	XX	XX	XX	XX	
**Expression of GOLPH3 in prostate**	High	XX	XX	XX	XX	XX	XX	XX	6	31.6	16	39.0	9	50.0	0.515
	Low	XX	XX	XX	XX	XX	XX		13	68.4	25	61.0	9	50.0	

IRS, immunoreactive scale; n, number; %, percentage; pT, pathological tumor stage; ECE, extracapsular extension; NVI, neurovascular invasion; LVI, lymphovascular invasion; efficacy of RP, defined as an PSA level <0.1 ng/ml at the first measurement after radical prostatectomy.

## 4 Discussion

PCa, the second most commonly diagnosed cancer in men, presents a serious diagnostic and therapeutic challenge for clinicians and pathologists. As life expectancy is increasing worldwide and PCa incidence is correlated with age, an increase in the number of men newly diagnosed with this type of cancer in the near future is expected (2). Nevertheless, despite significant advancements in adjuvant therapy resulting in increased cancer-specific survival, we still rely on classic factors such as PSA level, histological grade group, and clinical stage when establishing prognosis (31). Incorporating additional data such as IHC marker expression in postoperative specimens could improve patient prognosis after RP. Therefore, it is necessary to identify reliable biomarkers. The role and application of IHC biomarkers in the diagnosis and prognosis of PCa, including the formation of metastases, are the subject of many ongoing studies. Many of the results from these studies are promising but are not currently reflected in urological guidelines regarding PCa (13, 32). Although prostatic expression of GOLPH3 has been evaluated in several studies, to date, no study has examined the lymph node expression of this marker, which makes our research innovative (3, 4, 6, 7, 33).

According to our statistical analysis, GOLPH3 expression assessed using the IRS scale was higher in the material from the metastatic lymph node than from the prostate (IRS score: 8 vs. 6; p = 0,056), which may suggest that it plays a significant role not only in proliferation and cell cycle regulation, but also in the formation of distant metastases. Our study also found a positive correlation between the level of GOLPH3 expression in prostate tissue and metastatic lymph node tissue (rho = 0,294). Future lymph node examination of GOLPH3 expression might be a promising direction in tissue marker diagnostics, considering its increased nodal expression compared to that in prostate specimens. GOLPH3 involvement in metastasis has already been demonstrated in a study by Song et al. (34), where GOLPH3 overexpression correlated positively with clinicopathological characteristics, such as nodal status (p = 0.007), in patients with non-small-cell lung cancer (NSCLC). In the same study, the authors reported that NSCLC cells expressing GOLPH3 at high level injected into the tail vein of a mouse model presented higher metastatic capabilities than GOLPH3-silenced cells. Moreover, the migratory and invasive abilities of NSCLC cells were significantly higher in GOLPH3-overexpressing cell lines. We found a positive correlation between the level of GOLPH3 expression on both the IRS scale and the intensity of staining in the metastatic lymph nodes and the percentage of total lymph nodes with metastases. These results suggest that GOLPH3 may be an important factor in pre-metastatic niche formation; however, since correlation does not necessarily constitute causation, more data and biological proof are needed to prove this hypothesis. In the future, this observation may also be helpful in improving the models used to determine the risk of nodal metastases of PCa, such as the Briganti nomogram. This issue is extremely important in the decision-making process regarding the determination of the indications for lymphadenectomy, which significantly increases the risk of surgery and potential complications (12, 35–37).

GOLPH3 is also considered a negative prognostic factor in patients with PCa. In a study by El-Maqsoud et al., patients with high GOLPH3 expression had a higher Gleason score and disease stage. Moreover, moderate or intense marker levels were the sole predictors of overall survival (5). Overexpression of GOLPH3 was also associated with the transition of PCa from hormone-sensitive to hormone-resistant and shorter disease-free survival and overall survival (6). In our study, we found that LVI tended to be more common in patients with a higher percentage of GOLPH3-positive cells (p = 0.02). Although we failed to demonstrate a correlation between GOLPH3 expression level and oncological outcome, LVI was established as a negative prognostic factor in patients with PCa (38–40). We also found a positive association between the intensity of GOLPH3 staining in the metastatic lymph nodes and EAU classification (rho = 0.242), which was also highlighted in previous studies (3, 5). In the subject of the use of GOLPH3 expression assessment as a prognostic parameter, in our study, we found a significant correlation between GOLPH3 expression and efficacy of RP. The higher the percentage of GOLPH3 positive cancer cells (“A” score in IRS scale) in the prostate and the higher the staining intensity (“B” score in IRS scale) in the metastatic lymph node, the efficacy of RP was lower (p<0.05). We define efficacy of RP as a PSA level <0.1 ng/ml at the first measurement after RP, approximately 6 weeks after surgery (this value was also used to define so-called persistent PSA). Persistent PSA after RP occurs in 5–20% of patients and may result from various causes, including pre-existing metastases or residual benign prostate tissue (41, 42). Studies have shown that persistent PSA after RP is associated with more advanced disease characteristics (for example, higher pathologic stage, positive nodal status, or pathologic ISUP grade > 3) and poorer prognosis (worse 5-year biochemical recurrence-free survival and ten-years overall survival than patients without persistent PSA after RP) (43). Also, detectable PSA after RP (>0.1 ng/ml) significantly increases the risk of metastasis formation (44, 45). Although not all patients with persistent PSA experience disease recurrence (46), it remains a significant factor in predicting adverse oncological outcomes.

We are aware of the limitations of this study. Firstly, it was conducted on a relatively small group of patients, which could have resulted in a lack of statistical power to detect subtle differences and may have introduced bias. Secondly, there was an absence of follow-up data for patients who underwent RP. The lack of long-term data hampers our ability to evaluate the impact of the expression level of GOLPH3 on patient outcomes, such as biochemical recurrence (BCR) or overall survival. Thirdly, the expression of the GOLPH3 evaluation method employed in this study, the IRS scale, has certain limitations that require further improvement. To address this issue, future studies should consider the use of the H-score method (47, 48) (requiring more experience from the uropathologist, but allowing for a more detailed assessment of the material, taking into account even the heterogeneity of staining within one sample) as a method of assessing GOLPH3 expression in preparations. The last limitation of the study was that we tested GOLPH3 expression only in PCa tissue without comparison with the control group; for example, normal prostatic tissue adjacent to tumor cells obtained via biopsies, tissues of benign prostatic hyperplasia after transurethral resection of the prostate (TURP) or lymph nodes of patients after RP without metastases. However, the limitations we identified should not detract from the strengths of our study, which originate from its innovative nature and the rigorous methodology applied. The unique feature of our study is that it was the first time that GOLPH3 expression was tested in PCa metastatic lymph nodes; however, we see a need and plan to extend our study in the future with the above-described comparison to a control group. This will further define the role of GOLPH3 in PCa and its potential clinical implications.

## 5 Conclusions

GOLPH3 is expressed in both the prostate and metastatic lymph nodes, with higher expression in the metastatic lymph nodes (however, this difference was not statistically significant, p=0.056) and a positive correlation between GOLPH3 expression levels in the prostate and metastatic lymph nodes, suggesting a potential connection between primary and metastatic tumors. High GOLPH3 expression is associated with LVI, the percentage of all metastatic lymph nodes, and the high-risk group in the EAU classification, but there was no significant correlation between GOLPH3 expression levels and the other pathological features or postoperative outcomes of patients. Further research is needed to understand the functional significance and potential clinical applications of GOLPH3 in prostate cancer.

## Data availability statement

The raw data supporting the conclusions of this article will be made available by the authors, without undue reservation.

## Ethics statement

The studies involving humans were approved by Ethics Committee of Wroclaw Medical University. The studies were conducted in accordance with the local legislation and institutional requirements. The human samples used in this study were acquired from a by- product of routine care or industry. Written informed consent for participation was not required from the participants or the participants’ legal guardians/next of kin in accordance with the national legislation and institutional requirements.

## Author contributions

PK: Conceptualization, Investigation, Methodology, Writing – original draft. MK: Conceptualization, Investigation, Methodology, Validation, Writing – review & editing. KK: Resources, Writing – original draft. AP: Conceptualization, Methodology, Visualization, Writing – review & editing. ŁN: Data curation, Resources, Writing – review & editing. WK: Resources, Writing – review & editing. AG: Writing – review & editing. KD: Data curation, Writing – review & editing. PD: Methodology, Supervision, Writing – review & editing. AH: Methodology, Supervision, Writing – review & editing. TS: Project administration, Supervision, Writing – review & editing. BM: Conceptualization, Funding acquisition, Project administration, Resources, Supervision, Writing – review & editing.
